# The strong correlation between visual function improvement and retinal microcirculation enhancement in glaucoma

**DOI:** 10.3389/fmed.2025.1537741

**Published:** 2025-03-19

**Authors:** Ting Wang, Qiying Ling, Boyu Shen, Xu Jia

**Affiliations:** ^1^Department of Ophthalmology, Tongren People’s Hospital, Tongren, China; ^2^Department of Ophthalmology, The Affiliated Hospital of Guizhou Medical University, Guiyang, China; ^3^Department of Ophthalmology, Jinan University, Guangzhou, China

**Keywords:** glaucoma, vessel density, intraocular pressure, visual field, optical coherence tomography angiography

## Abstract

**Introduction:**

This study aimed to investigate the alterations in retinal vessel density (VD) among glaucomatous patients following effective intraocular pressure (IOP) reduction and to explore the relationship between retinal VD, visual function, and optic nerve structure.

**Methods:**

Participants diagnosed with primary open-angle glaucoma (POAG) and chronic primary angle-closure glaucoma (CPACG) were included. We measured peripapillary and macular VD, retinal nerve fiber layer (RNFL) thickness, foveal avascular zone (FAZ), and visual field (VF) parameters before treatment, and at 1 week, 1 month, 3 months, and 6 months post-treatment. The data were analyzed using ANOVA and Pearson correlation analysis.

**Results:**

A total of 20 patients were included. Significant improvements in peripapillary VD were observed in the superior and nasal sectors at 1 week, superior and temporal sectors at 1 month, and in the superior, inferior, and temporal sectors at 3 months, with sustained improvements in the superior, nasal, and temporal sectors at 6 months. Recovery of macular VD was noted across all sectors at 1 week, predominantly in the superior parafovea at 1 month, and in the superior, inferior parafovea, and inferior perifovea by 3 months, with further improvement in the inferior parafovea and perifovea at 6 months. The FAZ area significantly narrowed within the first 3 months. The mean deviation (MD) value demonstrated an increase at 1 week, 3 months, and 6 months. Notably, changes in peripapillary VD in the superior and inferior sectors exhibited a strong correlation with MD values, while correlations in the nasal and temporal sectors were moderate. Conversely, the correlation between IOP changes and MD was weak.

**Discussion:**

Effective IOP reduction was beneficial for the recovery of both peripapillary and macular microcirculation, leading to improvements in visual function, suggesting that actively improving retinal microcirculation while reducing IOP may contribute to partial recovery of visual function for patients with chronic glaucoma.

## Introduction

Glaucoma is one of the leading causes of irreversible blindness and visual impairment, characterized by structural damage to the optic nerve and associated visual dysfunction, with elevated intraocular pressure (IOP) identified as the principal pathogenic factor. Clinical observations suggest that the mechanical pressure theory, which attributes the pathogenesis and recovery post-treatment in certain glaucomatous patients to elevated IOP, is inadequate (1). Consequently, the vascular ischemia theory has been proposed, positing that retinal vascular hypoperfusion or dysregulation—whether primary or secondary to ocular hypertension—can diminish the tolerance of retinal ganglion cells (RGCs) to IOP. This may expedite the progressive apoptosis and necrosis of RGCs, ultimately leading to thinning of the retinal nerve fiber layer (RNFL) and structural remodeling (2, 3). Research has demonstrated that ocular perfusion pressure and peripapillary vessel density (VD) are reduced in various forms of chronic glaucoma compared to healthy subjects, with a noted reduction in retinal vascular density occurring prior to optic nerve structural damage (4–6). However, current investigations into glaucomatous microvasculature predominantly focus on cross-sectional comparisons between affected individuals and healthy controls, lacking longitudinal evidence regarding microvascular changes and the relationships between alterations in blood flow, optic nerve structure, and function following effective target-IOP treatment. Optical coherence tomography angiography (OCT-A) has emerged as a valuable tool due to its non-invasive nature, rapid acquisition, repeatability, intuitiveness, and ability to provide layered quantitative analysis. In this study, we utilized OCT-A to assess trends in peripapillary and macular VD in primary chronic glaucomatous eyes before and after standard IOP-lowering treatment. Our objective was to explore the associations between changes in blood flow density and optic nerve structure and function, thereby underscoring the significance of retinal microcirculation in the prognosis of the glaucomatous population.

## Materials and methods

This prospective observational study was conducted in the ophthalmology clinic of the Affiliated Hospital of Guizhou Medical University. Written informed consent was obtained from each participant.

### Study population

Participants diagnosed with primary open-angle glaucoma (POAG) and chronic primary angle-closure glaucoma (CPACG) (7–9) between November 2020 and September 2021 were prospectively enrolled based on predefined inclusion and exclusion criteria. Inclusion criteria included: (1) IOP >25 mmHg at the initial visit; (2) effective attainment of target IOP (<15 mmHg) after medical or surgical intervention, maintained for over 6 months; (3) spherical refraction ranging from −6.00 to +6.00 diopters, cylindrical refraction ranging from −3.00 to +3.00 diopters, and best-corrected visual acuity (BCVA) >0.1; (4) age >18 years; (5) clear optical structures. Exclusion criteria comprised: (1) secondary glaucoma, high myopia, retinopathy, or other ophthalmic diseases; (2) initial IOP <25 mmHg or >50 mmHg; (3) histories of hypertension, diabetes mellitus, neurological disorders, or systemic vascular diseases; (4) total visual field blindness; (5) poor compliance or loss to follow-up; (6) patients with unclear refractive media, such as corneal edema or lens opacity; (7) OCT-A signal strength less than 8; (8) the symptoms of primary acute angle-closure glaucoma or acute exacerbation of primary chronic angle-closure glaucoma include eye congestion, eye pain, and central vision loss. (9) During the follow-up period, cataract or other ophthalmic surgeries were performed. If the maximum medical therapy could not achieve target IOP within 2 weeks, trabeculectomy was considered. To minimize the potential impact of medications on retinal VD in our study, for surgical patients, PGs and β-blockers should be discontinued 1 week prior to surgery. After trabeculectomy, all surgical patients should stop using any anti-glaucoma medications. Similarly, for non-surgical patients, PGs and β-blockers should also be discontinued, and patients who are allowed to switch to carbonic anhydrase inhibitors (such as brinzolamide) and/or α-agonists (such as brimonidine tartrate) to achieve the target IOP are included in the study.

All eligible patients underwent comprehensive ophthalmic assessments, including slit-lamp biomicroscopy, measurement of BCVA, IOP (using Goldmann applanation tonometry), central corneal thickness (CCT) via Pentacam (Germany, Oculus), gonioscopy, Humphrey visual field (VF) examination (Carl Zeiss Meditec), optical coherence tomography (OCT) examination (evaluating macular and optic disc structures as well as RNFL thickness), and OCT-A (Carl Zeiss Meditec) imaging of the peripapillary and macular regions at the initial visit. Follow-up assessments were conducted at 1 week, and at 1, 3, and 6 months post-treatment, including repeated slit-lamp biomicroscopy, BCVA, IOP, OCT, OCT-A, and VF examinations. All assessments were performed by the same skilled ophthalmologist who was not involved in the study design or data collection.

### OCT-A image acquisition

Vascular images of the macula and optic nerve head (ONH) were obtained using an OCT-A device (Carl Zeiss Meditec) by a single experienced technician throughout the study. Peripapillary images were captured in 3D Angio Disc mode, with a 4.5 × 4.5 mm auto-tracking field of view. Radial peripapillary capillary (RPC) segmentation was defined as extending 70.2 μm from the surface to below the inner limiting membrane (ILM), while the peripapillary retina was delineated as a ring area of 0.75 mm extending outward from the optic disc boundary. Imaging of the superficial capillary plexus (SCP), situated between the ILM and the inner plexiform layer (IPL), was performed using 6.0 × 6.0 mm continuous scans in 3D Angio Retina mode. The boundaries of the foveal avascular zone (FAZ) were manually adjusted.

### OCT-A image processing

**Figure 1:**
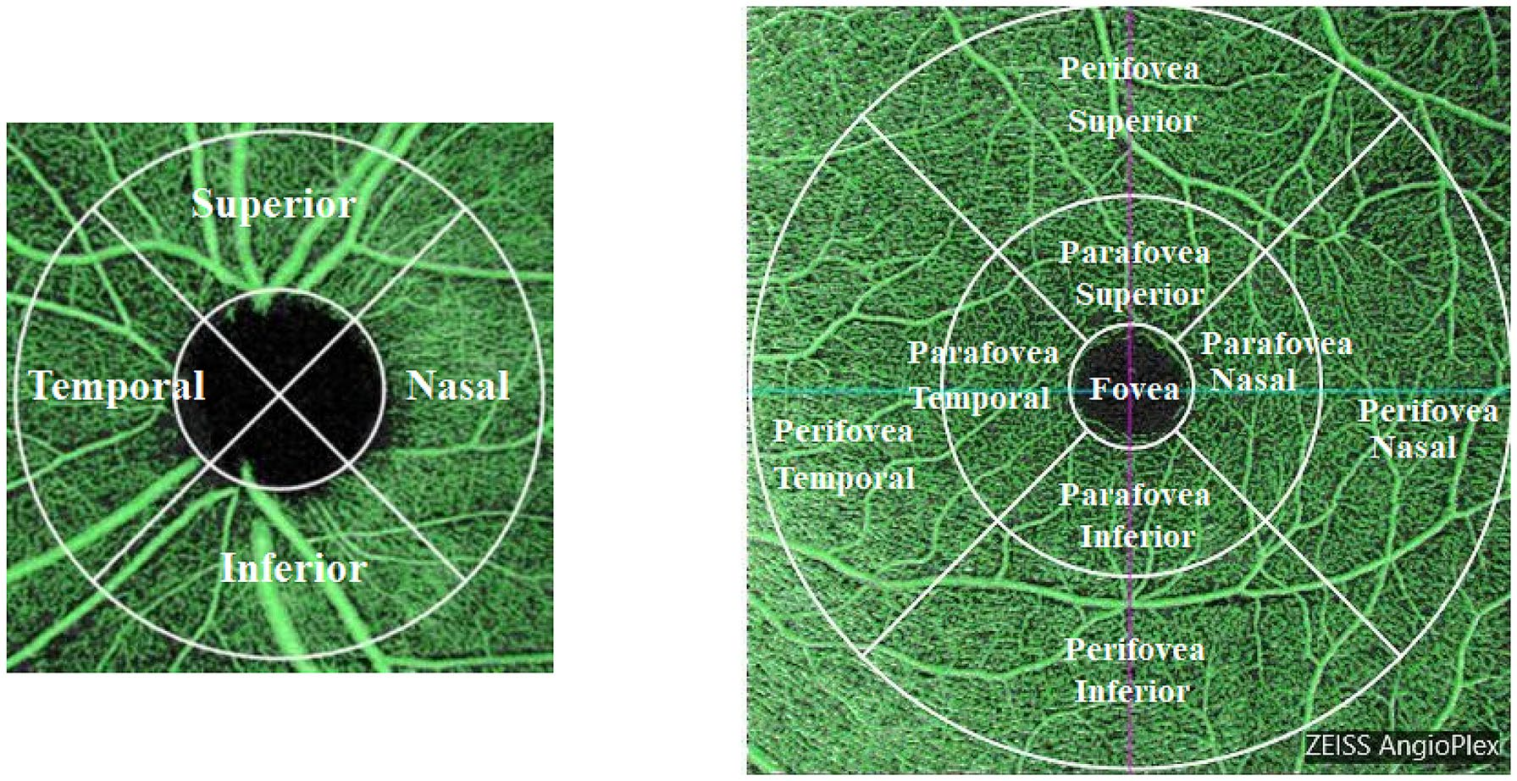
Scan regions for peripapillary and macular vessel density (VD). The left panel illustrates the peripapillary VD regions, categorized into superior, inferior, temporal, and nasal quadrants surrounding the optic nerve head. The right panel depicts the macular VD regions, segmented into concentric zones: fovea, parafovea, and perifovea, each further divided into superior, inferior, temporal, and nasal quadrants.

The vascular images from the SCP and RPC layers were directly exported from the OCT-A device database. For the peripapillary images, an inner circle corresponding to the optic disc edge and an outer circle defining the peripapillary retina were created using Adobe Photoshop software (version 2020) to facilitate subsequent calculations. The images were divided into four quadrants (superior, nasal, inferior, and temporal) along the square diagonal line (Figure 1). The percentage of vascular area, non-vascular area, and vessel density (VD) were quantified using Image J software. Macular VD measurements were obtained directly via the OCT-A’s CIRRUS AngioPlex quantitative analysis software, following the ETDRS regional settings (Figure 1). The FAZ area (mm^2^) was automatically mapped and measured by this software. The ONH was segmented in both Optic Disc Cube 200 × 200 and ring scanning modes to determine the vertical cup-disc ratio (C/D) and RNFL thickness values.

### Visual field examination

Following correction of refractive error, patients underwent visual field (VF) assessment using the Humphrey Visual Field Analyzer (Carl Zeiss Meditec) employing the 24-2 pattern with the Swedish Interactive Threshold Algorithm standard strategy. Reliable VF results were defined by a fixation loss rate of <20%, a false positive rate of <15%, and a false negative rate of <15%. Participants with poor reliability were required to repeat the post-learning examination until the reliability criteria were met. The mean deviation (MD) and pattern standard deviation (PSD) values were subsequently analyzed statistically.

### Intraocular pressure measurement

IOP was measured using Goldmann applanation tonometry at 10 a.m., and the mean of three measurements was recorded.

### Statistical analysis

Statistical analysis was conducted using IBM SPSS Statistics for Windows (Version 25.0, IBM Corp., Armonk, NY). Following the Shapiro–Wilk test for normality, quantitative data conforming to a normal distribution were expressed as mean and standard deviation (SD). Repeated measures analysis of variance (ANOVA) was utilized to compare values between pre-treatment and subsequent follow-up visits. Bonferroni’s multiple comparison test was performed following the repeated measures ANOVA. All statistical tests were two-sided, with a *p*-value of <0.05 considered statistically significant. Pearson correlation analysis was employed to assess the correlations between peripapillary and macular VD, FAZ, RNFL thickness, C/D ratio, and MD values across different planned visits. The strength of correlation was categorized as strong (*r* ≥ 0.6), moderate (0.6 > *r* ≥ 0.4), and weak (*r* < 0.4).

## Results

In this study, a total of 20 patients (20 eyes) were included in the final analysis, comprising 11 males (55%) and 9 females (45%), with a mean age of 58.85 ± 9.52 years. Among the 20 patients, 9 were diagnosed with primary open-angle glaucoma (POAG) and 11 with chronic primary angle-closure glaucoma (CPACG). IOP control was achieved in 11 patients using anti-glaucoma medications and in 9 patients through trabeculectomy (without postoperative anti-glaucoma medications). Due to the shared characteristics of chronic elevated IOP, slow progression, similar vascular perfusion profiles, and absence of optic disc edema in both POAG and CPACG, we analyzed the groups together. The duration of medication prior to trabeculectomy ranged from a minimum of 2 weeks to a maximum of 1 year. None of the CPACG patients underwent laser peripheral iridotomy (LPI) and none continued using anti-glaucoma medications post-trabeculectomy. Detailed information regarding the types and duration of medications used prior to surgery for each surgical patient is provided in Supplementary Table 1, while Supplementary Table 2 outlines the medications for the medically treated patients. All patients reported a history of decreased vision or ocular discomfort lasting from 1 week to 3 months, although IOP was not monitored during this period. The mean best-corrected visual acuity (BCVA) before treatment was 0.6 (range: 0.4 to 0.8), and the mean central corneal thickness (CCT) was 528.50 ± 18.28 μm. Table 1 summarizes the clinical characteristics of the participants.

**Table 1:** Demographics and ocular characteristics of the study population (*N* = 20).

Characteristics	Mean ± SD
Age^a^ (years)	58.85 ± 9.52
Gender (male/female)	11/9
Laterality (OD/OS)	10/10
Central cornea thickness^a^ (CCT) (μm)	528.50 ± 18.28
Gonioscope (closure angle/open angle)	9/11
Treatment (trabeculectomy/medicine)	9/11
Best corrected visual acuity^b^ (BCVA)	0.60 (0.40, 0.80)

a Normally distributed variables, results are shown as mean ± SD.

b Non-normally distributed variables, results are shown as M (P25, P75).

### IOP reduction

**Figure 2:**
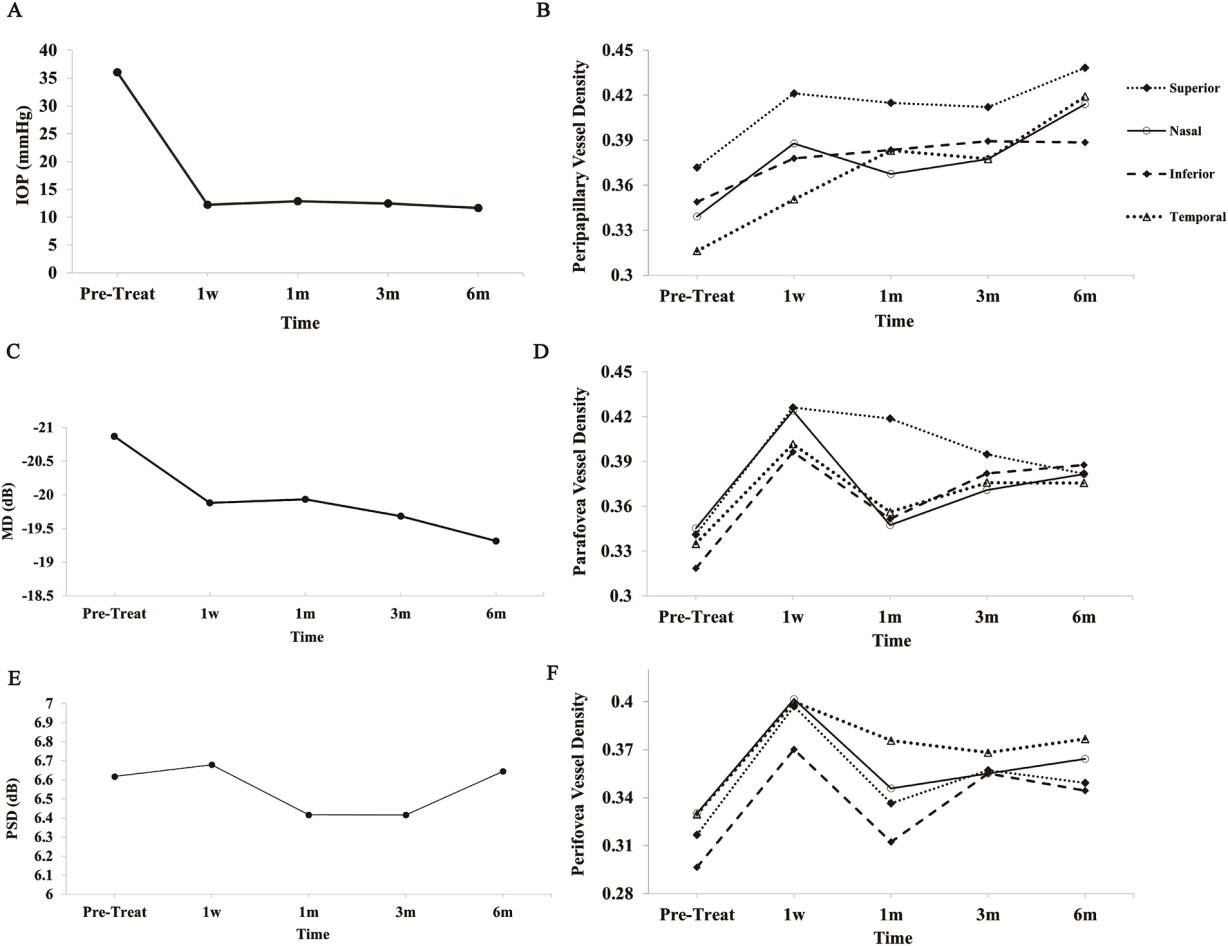
Changes in optic nerve structure and function following treatment. **(A)** IOP decreased significantly post-treatment and remained stable at 1 week, 1 month, 3 months, and 6 months. **(B)** Peripapillary VD in the superior, inferior, nasal, and temporal sectors displayed distinct trends, with gradual increases noted in some regions. **(C)** MD values from visual field testing improved following treatment. **(D)** Parafoveal VD across the superior, inferior, nasal, and temporal quadrants demonstrated longitudinal variations. **(E)** PSD values exhibited minor changes with initial fluctuations, ultimately stabilizing over time. **(F)** Perifoveal VD also exhibited dynamic changes across the superior, inferior, nasal, and temporal sectors. IOP, intraocular pressure; VD, vessel density; MD, mean deviation; PSD, pattern standard deviation.

The mean IOP decreased significantly from 36.05 ± 9.23 mmHg to the targeted IOP of approximately 12 mmHg by 1 week post-treatment, remaining stable through to the 6-month follow-up (*p* < 0.01). The average percentage reduction in IOP exceeded 60% (Figure 2A and Table 2).

**Table 2:** Changes in IOP, MD, PSD, C/D, and RNFL thickness parameters of pre-and post-treatment.

	Pre-treat	1-week post-treat	1-month post-treat	3-month post-treat	6-month post-treat	*F*
IOP (mmHg)	36.05 ± 9.23	12.25 ± 3.27^**^	12.90 ± 2.01^**^	12.46 ± 2.02^**^	11.65 ± 2.15^**^	27.968
MD (dB)	−20.87 ± 9.39	−19.88 ± 9.39^*^	−19.93 ± 9.33	−19.69 ± 9.13^***^	−19.31 ± 9.15^**^	3.113
PSD (dB)	6.62 ± 3.46	6.68 ± 3.29	6.42 ± 3.2	6.37 ± 2.73	6.56 ± 2.96	0.251
C/D	0.81 ± 0.09	0.80 ± 0.08	0.81 ± 0.08	0.80 ± 0.09	0.80 ± 0.08	0.476
RNFL (μm)	69.00 ± 12.96	66.45 ± 10.39	65.95 ± 10.21	65.70 ± 10.14	66.70 ± 10.36	1.048

IOP, intraocular pressure; MD, mean deviation; PSD, pattern standard deviation; C/D, cup-disc ratio; RNFL, retinal nerve fiber layer; pre-treat, pre-treatment; post-treat, post-treatment. ^*^p < 0.05, ^**^p < 0.01, ^***^p < 0.001. *p*-value represents the statistical significance of the differences in the mean measurements between each visit post-treatment and pre-treatment as calculated by repeated measures analysis of variances (ANOVA).

### Structural changes of optic nerve

The mean cup-to-disc ratio (C/D) (Supplementary Figure 1A and Table 2) and RNFL thickness (Supplementary Figure 1B and Table 2) at various follow-up visits showed no statistically significant differences compared to pre-treatment values (*p* > 0.05).

### Vessel densities

#### Peripapillary vessel density

**Figure 3:**
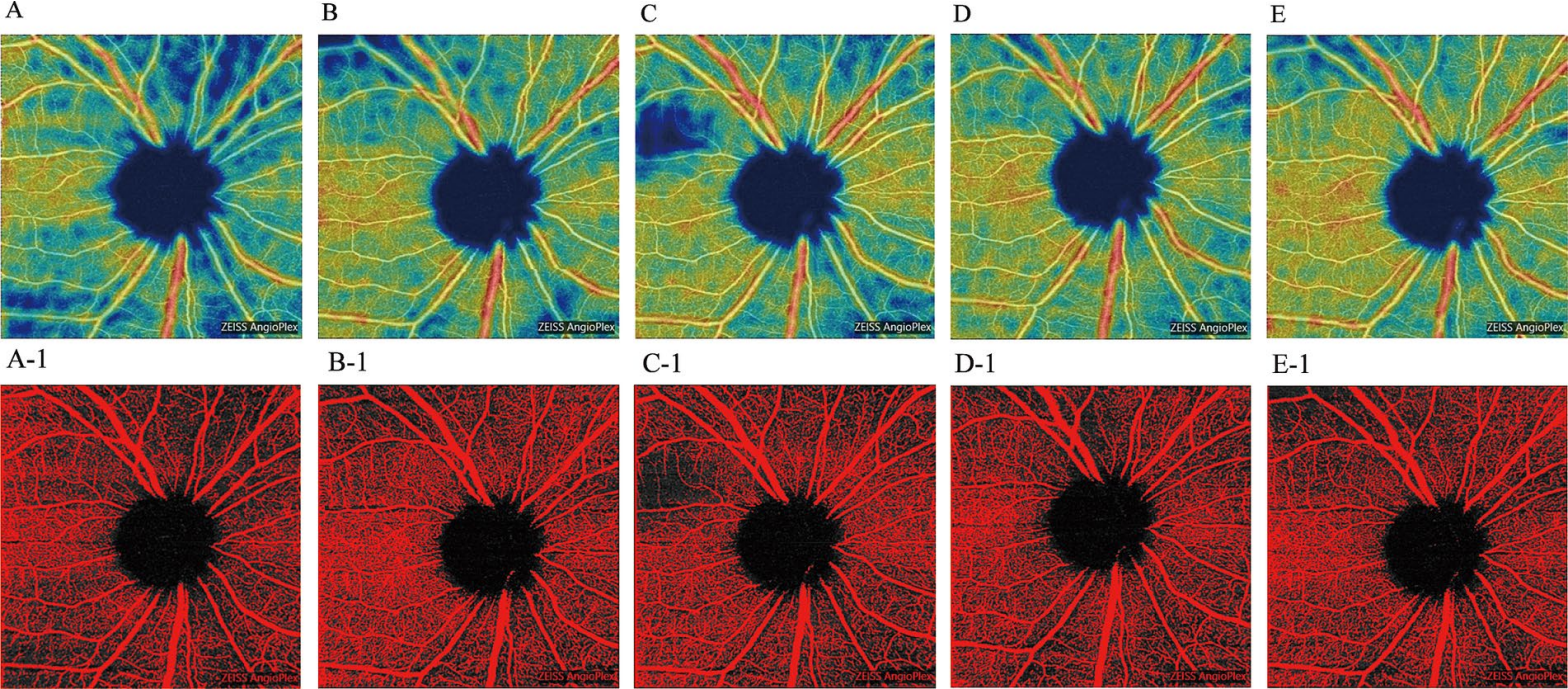
OCT-A images of the peripapillary VD in the right eye of a male patient, shown pre-treatment and during the 6-month follow-up period. (**A**: pre-treatment; **B**: 1-week post-treatment; **C**: 1-month post-treatment; **D**: 3-month post-treatment; **E**: 6-month post-treatment). Corresponding threshold maps **(A-1–E-1)**, generated using Image J, highlight the peripapillary VD in red.

Peripapillary VD was assessed using the OCT-A device. Following effective IOP control, peripapillary VD across all quadrants exhibited a general increasing trend. Notably, significant increases in superior VD were observed at 1 week, and in temporal VD at 1 month post-treatment compared to pre-treatment values (*p* < 0.05). Similarly, nasal VD showed significant improvements at 1 week and again at 6 months post-treatment (*p* < 0.05), although it experienced a slight reduction at 1 month before rising again at 3 months. A statistically significant increase in inferior VD was noted only at the 3-month follow-up (*p* < 0.05) (Figure 2B and Table 3). We present OCT-A images of peripapillary VD for one patient (right eye, male) before treatment and at the 6-month follow-up (Figure 3). These images illustrate the initial low peripapillary VD and weak blood flow signal before treatment (Figures 3A,A-1), with gradual increases in both VD and blood flow signal observed at the 6-month follow-up (Figures 3B–E,B-1–E-1).

**Table 3:** Changes in peripapillary and superficial macular vessel density and foveal avascular zone parameters pre-and post-glaucoma surgery.

	Pre-treat	1-week post-treat	1-month post-treat	3-month post-treat	6-month post-treat	*F*
Peripapillary						
Superior	0.37 ± 0.12	0.42 ± 0.12^***^	0.42 ± 0.12^*^	0.41 ± 0.13^*^	0.44 ± 0.13^**^	5.07
Nasal	0.34 ± 0.11	0.39 ± 0.10^*^	0.37 ± 0.12	0.38 ± 0.12	0.41 ± 0.13^**^	3.22
Inferior	0.35 ± 0.10	0.38 ± 0.11	0.38 ± 0.11	0.39 ± 0.09^*^	0.39 ± 0.11	1.56
Temporal	0.32 ± 0.10	0.35 ± 0.11	0.38 ± 0.10^**^	0.38 ± 0.10^*^	0.42 ± 0.09^***^	7.22
Parafovea						
Superior	0.34 ± 0.11	0.43 ± 0.04^**^	0.42 ± 0.15^*^	0.40 ± 0.06^*^	0.38 ± 0.07	7.22
Nasal	0.35 ± 0.09	0.42 ± 0.04^**^	0.35 ± 0.08	0.37 ± 0.06	0.38 ± 0.06	8.30
Inferior	0.32 ± 0.12	0.40 ± 0.07^*^	0.35 ± 0.07	0.38 ± 0.06^*^	0.39 ± 0.07^*^	5.75
Temporal	0.34 ± 0.11	0.40 ± 0.07^*^	0.36 ± 0.07	0.38 ± 0.10	0.38 ± 0.08	2.45
Perifovea						
Superior	0.32 ± 0.11	0.40 ± 0.06^*^	0.34 ± 0.05	0.36 ± 0.06	0.35 ± 0.06	8.51
Nasal	0.33 ± 0.10	0.40 ± 0.06^**^	0.35 ± 0.09	0.36 ± 0.08	0.36 ± 0.07	7.83
Inferior	0.30 ± 0.11	0.37 ± 0.07^*^	0.31 ± 0.08	0.36 ± 0.07^**^	0.35 ± 0.08^*^	6.97
Temporal	0.33 ± 0.12	0.40 ± 0.09^*^	0.38 ± 0.07	0.37 ± 0.10	0.38 ± 0.09	4.31
FAZ (mm^2^)	0.22 ± 0.06	0.21 ± 0.07^*^	0.21 ± 0.07^*^	0.21 ± 0.06^**^	0.22 ± 0.07	6.88

FAZ, foveal avascular zone. ^*^p < 0.05, ^**^p < 0.01, ^***^p < 0.001. *p*-value represents the statistical significance of the differences in the mean measurements between each visit post-treatment and pre-treatment as calculated by repeated measures analysis of variances (ANOVA).

#### Macular vessel density

##### Parafovea region

In the early stage (1 week) after achieving target IOP, mean VD in each quadrant of the parafovea peaked but subsequently exhibited varying degrees of decline. By the 3-month follow-up, VD in most quadrants (excluding the superior quadrant) showed marked increases again. In contrast, the superior quadrant continued to display a decreasing trend. At 6 months post-treatment, all quadrants had significantly higher VD compared to pre-treatment values. Statistical analysis revealed significant increases in VD for the superior (except at 6 months) and inferior quadrants (except at 1 month) at each follow-up visit compared to baseline (*p* < 0.05). However, in the temporal and nasal sectors, only the VD measured at 1 week post-treatment was statistically significant compared to pre-treatment (Figure 2D and Table 3).

##### Perifovea region

**Figure 4:**
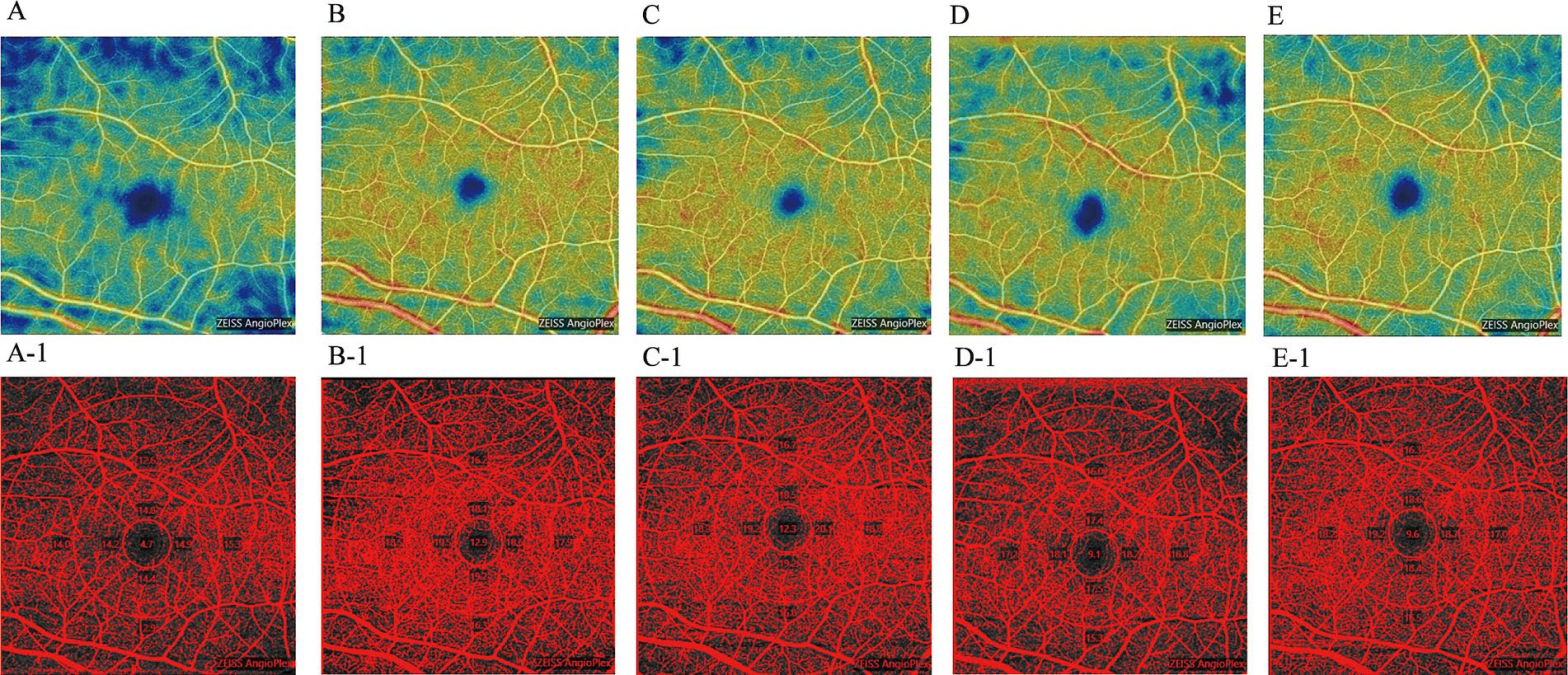
OCT-A images of the macular VD in the right eye of a male patient, shown pre-treatment and during the 6-month follow-up period. (**A**: pre-treatment; **B**: 1-week post-treatment; **C**: 1-month post-treatment; **D**: 3-month post-treatment; **E**: 6-month post-treatment). Corresponding threshold maps **(A-1–E-1)**, generated using Image J, highlight the macular VD in red.

In the immediate post-operative period, significantly elevated mean VD was observed in each quadrant of the perifovea region. VD in all quadrants peaked at 1 week but subsequently declined, particularly in the superior, inferior, and nasal quadrants. After 1 month, VD averages in all quadrants, except the temporal sector, gradually increased again. By the 3-month follow-up, an upward trend in VD was noted in the temporal and nasal sectors, while a downward trend was observed in the superior and inferior sectors compared to pre-treatment values. At the 6-month follow-up, VD in all sectors increased relative to pre-treatment levels. Statistical analysis indicated significant improvements in microvascular perfusion across all perifovea quadrants at 1 week post-treatment (*p* < 0.05). Conversely, only the inferior sector at 3 and 6 months showed significant differences compared to pre-treatment, while the other quadrants and time points did not (*p* > 0.05) (Figure 2F and Table 3). We also present OCT-A images of macular VD for one patient (left eye, male) before treatment and at the 6-month follow-up (Figure 4). These images demonstrate low macular VD and weak blood flow signal prior to treatment (Figure 4A,A-1). At 1 week, both VD and blood flow signal increased rapidly (Figure 4B,B-1), but subsequently decreased at 1 month (Figure 4C,C-1). After 3 months, VD and blood flow signal showed a gradual upward trend again (Figures 4D,E,D-1,E-1).

### Foveal avascular zone

With the effective reduction of IOP, the mean FAZ area across all recruited eyes showed a significant decrease at the early stage (1 week) and continued to decline up to 3 months, although the rate of decline was slower at the 1-month and 3-month marks. After 3 months, however, the FAZ area exhibited an increasing trend. Significant changes in the FAZ area were observed at 1 week, 1 month, and 3 months post-treatment (*p* < 0.05), but no statistically significant differences were noted at the 6-month follow-up compared to pre-treatment values (*p* > 0.05) (Supplementary Figure 1C and Table 3).

### Visual field

Following effective IOP treatment, the mean overall visual field mean deviation (MD) value for the 20 included patients showed a notable increase, while the mean pattern standard deviation (PSD) value decreased at 1 month and 3 months post-treatment, before reverting. In 15 of the 20 eyes, MD values exhibited a continuous rise over the 6-month follow-up, whereas the remaining 5 patients experienced fluctuations during the follow-up visits (Figure 2C and Table 2). Significant changes in MD values were observed at the 1-week, 3-month, and 6-month follow-ups (*p* < 0.05). However, no significant changes in PSD values were detected compared to baseline measurements (*p* > 0.05) (Figure 2E and Table 2).

### Correlation analysis between peripapillary and macular VD and MD

The preceding results indicated that the MD values of the patients increased to some extent following treatment. To explore the underlying mechanisms, we analyzed the correlations between changes in peripapillary VD and MD, changes in macular VD and MD, as well as changes in IOP and MD. Statistical analysis revealed that changes in peripapillary VD in the superior and inferior sectors were strongly correlated with changes in MD (*r* > 0.6), while changes in nasal and temporal VD demonstrated a moderate correlation with MD values (0.4 < *r* < 0.6). In contrast, the correlations between IOP changes and VD changes in each sector of the perifovea and parafovea were weak (*r* < 0.4). Similarly, a weak correlation was found between changes in IOP and MD (*r* < 0.4) (Table 4).

**Table 4:** Correlation analysis between peripapillary and macular vessel density and MD.

Pearson	*r*	*p*-value
Peripapillary		
Superior	**0.72** ^b^	**0.00**
Nasal	**0.59** ^a^	**0.00**
Inferior	**0.64** ^b^	**0.00**
Temporal	**0.51** ^a^	**0.00**
Parafovea		
Superior	−0.06	0.54
Nasal	−0.08	0.43
Inferior	−0.06	0.59
Temporal	−0.16	0.12
Perifovea		
Superior	−0.08	0.42
Nasal	−0.10	0.32
Inferior	−0.13	0.20
Temporal	−0.05	0.66
IOP	−0.11	**0.29**

a Moderate correlation.

b Strong correlation.Bold values: Data with positive results.

## Discussion

Glaucoma is an optic neuropathy characterized by progressive loss of retinal ganglion cells (RGCs), leading to irreversible visual impairment. Recent studies have established that systemic and localized ocular circulation insufficiencies, or vascular dysfunction, contribute to the development of glaucomatous optic neuropathy, either alone or in conjunction with elevated intraocular pressure (IOP) (10–12), furthermore, vessel density (VD) in the peripapillary and macular regions of primary open-angle glaucoma (POAG) patients is significantly reduced compared to normal subjects (13). This reduction in VD correlates with the severity of structural damage and dysfunction in the optic nerve (14), indicating that VD metrics may play a valuable role in glaucoma staging. However, many current studies are cross-sectional comparisons between glaucoma and normal subjects or among different types of glaucoma, lacking longitudinal evidence on the trends in VD changes with effective IOP lowering and the relationship between VD, visual function, and optic nerve structure.

### Vessel density

In this study, we found that after effective IOP reduction, superficial peripapillary vessel density (VD) exhibited an overall increasing trend over the 6-month follow-up period. Notably, peripapillary VD in each quadrant showed the most significant increase during the first week. However, in some quadrants, VD increased slowly or even decreased at the 1-month and 3-month marks, before rising significantly again at 6 months. These results suggest that effective IOP lowering can markedly improve superficial peripapillary microcirculation. There are both similarities and differences among existing research. Some studies reported significant increases in peripapillary VD in glaucoma patients at 3 and 6 months, with no statistical changes or even decreases noted at 1 week and 1 month (15, 16). Conversely, other researchers found no significant changes within a 6-month follow-up period (17–19). Furthermore, we observed that patients with significant increases in peripapillary VD had higher baseline IOPs (30.92 ± 6.32 mmHg and 24.5 ± 10.4 mmHg, respectively), while those without changes had lower baseline IOPs (19.4 ± 7.0 mmHg, 22.4 ± 2.4 mmHg, and 21.0 (17.07, 23.87) mmHg, respectively). Importantly, some studies indicated that patients with higher baseline IOP values and greater IOP reductions experienced more pronounced enhancements in peripapillary VD after treatment (20). In our study, the mean baseline IOP was 36.05 ± 9.23 mmHg, with reductions exceeding 60%, and the observed changes in peripapillary vascular regulation aligned with previous findings. Thus, we conclude that effective IOP lowering is particularly beneficial for glaucoma patients with higher baseline IOP values in terms of peripapillary microcirculatory recovery. We theorize that variations in race and the autoregulatory capabilities of blood vessels may contribute to the differing fluctuations in peripapillary VD observed across studies, although the overall trend remains upward.

This study found that after effective IOP lowering, vessel density (VD) in each quadrant of the parafovea and perifovea significantly increased at the early stage (1 week), decreased at 1 month, re-increased at 3 months, and tended to stabilize at 6 months, although some quadrants exhibited a downward trend again. These results suggest that effective IOP reduction can significantly enhance superficial macular microcirculation in the early stage, but the improvement may not be optimal after 6 months. Findings from previous studies regarding macular VD changes have been inconsistent. Some studies reported increases at 1 month and 6 months (16, 19), while others noted a decrease at 12 months (17). Additionally, our results indicate that microvascular recovery in the macular region appears to be less pronounced than in the peripapillary region, consistent with prior findings (16). It has been suggested that peripapillary VD is more meaningful for assessing optic nerve function in glaucoma (21). Importently, current literature clearly indicates that carbonic anhydrase inhibitors (such as brinzolamide) and/or α-agonists (such as brimonidine tartrate) have been shown to have no significant impact on retinal microvascular density (22–24), suggesting that these medications effectively lower intraocular pressure (IOP) without altering retinal vessel density. To minimize the potential impact of medications on retinal VD in our study, for surgical patients, PGs and β-blockers should be discontinued 1 week prior to surgery. After trabeculectomy, all surgical patients should stop using any anti-glaucoma medications. Similarly, for non-surgical patients, PGs and β-blockers should also be discontinued, and patients who are allowed to switch to carbonic anhydrase inhibitors (such as brinzolamide) and/or α-agonists (such as brimonidine tartrate) to achieve the target IOP are included in the study.

However, the mechanisms underlying the different responses of macular and peripapillary microvascular circulation to IOP changes remain unclear. One study indicated that reduced posterior displacement in the lamina cribrosa (LC) and prelaminar tissue following glaucoma treatment was associated with increased VD in the LC, which correlated more strongly with reductions in LC curvature than with IOP decline (25, 26). This finding suggests that the reduction in LC curvature due to IOP lowering could relieve vascular compression within the LC, potentially enhancing perfusion to the optic nerve head (ONH) and peripapillary regions (27). Alternatively, we propose a different hypothesis. The blood supply to the ONH has its own autonomic regulatory mechanisms that maintain perfusion through myogenic and metabolic feedback. When perfusion pressure fluctuates within a certain range, the terminal microvessels adjust blood flow resistance by modulating their contraction and relaxation, thus achieving autonomic regulation of local blood flow and maintaining relatively stable perfusion. In glaucoma patients, this regulatory mechanism may be impaired or absent, leading to local blood supply disorders and further deterioration of optic nerve function. Moreover, IOP reduction alleviates compression of the central retinal artery, initially increasing peripapillary and macular VD through vasodilation from enhanced passive blood supply. This is supported by our observation of the most significant VD increase at the early stage (1 week) post-treatment. Subsequently, the vascular autonomic regulatory mechanisms in the peripapillary and macular areas may gradually recover, albeit at a lower level, leading to a decrease in VD at 1 month post-treatment. As the vascular autonomic regulatory mechanisms continue to restore, blood volume in the vasculature gradually increases, which is evidenced by the re-increase in VD at 3 and 6 months. The differences in microcirculatory recovery between the peripapillary and macular areas may be due to the superficial capillary plexus (SCP) being farther from the central retinal artery and thinner than peripapillary vessels. This makes it more susceptible to compression from high IOP, resulting in a slower recovery of autonomic regulatory function, and potentially leading to dysfunction in some vessels after perfusion is restored. Therefore, we conclude that the reduction in IOP and changes in vascular density in the peripapillary and macular regions have a promotive effect on the improvement of visual function. This suggests that, in future clinical practice, follow-up assessments of glaucoma patients under effective treatment should not only focus on changes in intraocular pressure but also pay particular attention to changes in vascular density in the peripapillary and macular regions.

### Foveal avascular zone

The FAZ serves as a capillary-free zone in the macular region, reflecting macular microcirculation to some extent. Ch’ng et al. (17) reported a significant enlargement of the FAZ area 1 month after surgery, which gradually returned to baseline levels by 12 months. They suggested that this enlargement was due to surgical inflammation in the early postoperative period. In contrast, Shoji et al. (28) observed a narrowing of the FAZ area over a 3-month follow-up, hypothesizing that some inner capillary blood vessels, below the detection threshold, became visible only after microcirculation recovery. In our study, the trend of the FAZ area was consistent with changes in macular VD following effective IOP reduction. We believe that the partial increase in blood supply to the macular area led to thickening of surrounding tissue and some functional recovery, indicating that changes in the FAZ area can effectively evaluate macular microcirculation.

### Visual field and structure in optic nerve

Visual field (VF) examination is crucial for glaucoma staging and disease evaluation during follow-up. Previous studies have shown correlations between peripapillary VD parameters, glaucoma severity, and MD values (13). Specifically, a decrease in peripapillary VD correlates significantly with the severity of VF defects, with a decline of 0.66 dB in MD for every 1% decrease in peripapillary VD, and a decline of 0.639 dB for every 1% decrease in total VD in the macular superficial capillary plexus (29, 30). In our study, we found that the mean overall MD value of primary chronic glaucoma patients significantly increased after effective IOP control, particularly at 1 week and 6 months. The changes in MD were consistent with the trends observed in peripapillary VD. Furthermore, a strong to moderate correlation was established between the VD of each peripapillary quadrant and MD values, indicating a close relationship between peripapillary VD and visual function. We observed a weak correlation between IOP reduction and MD, suggesting that IOP reduction may act as a dynamic factor in improving peripapillary and macular microcirculation, further promoting visual recovery. This finding indirectly supports the notion that glaucoma may be an optic neuropathy associated with retinal vascular disorders, and enhancing retinal microcirculation could have a protective role for the optic nerve.

Current studies suggest that adult glaucoma patients may not experience significant structural recovery in the optic nerve head (ONH) even after IOP reduction (31). In our study, no statistical changes were noted in the cup-to-disc (C/D) ratio or RNFL thickness, consistent with prior reports. Many clinicians contend that structural damage in the optic nerve leads to visual dysfunction, reflecting the difficulty of achieving visual restoration solely through IOP control (32). Although we observed no significant structural changes in the optic nerve with effective IOP lowering, visual function showed partial recovery. This indicates that visual defects do not strictly correspond to optic nerve structure during the recovery phase of the disease. Visual field impairment in glaucoma patients in the early stages arises primarily from reductions in the dendritic field and soma of retinal ganglion cells (RGCs), especially magnocellular RGCs. Based on this understanding, we speculate that RGCs experience partial degeneration and apoptosis rather than complete necrosis under elevated IOP, resulting in partial inhibition of visual function (33). With effective IOP reduction and localized vascular circulation improvement, the function of inhibited neurons may partially recover, leading to some degree of visual improvement (34–36). Conversely, changes in optic nerve structure represent the severity of RGCs dysfunction and damage, suggesting that partial visual function recovery may not coincide with IOP reduction or increased VD, explaining the observed partial improvement in MD values alongside no significant changes in PSD values.

Previous results suggest that microcirculation-enhancing therapies may play a positive role in promoting visual function in glaucoma patients. However, these therapies have not been considered primary treatments in current glaucoma management guidelines across various countries. A limited number of studies have indicated that improving microcirculation can facilitate disease recovery in glaucoma patients, particularly those with normal-tension glaucoma accompanied by systemic or localized vascular dysfunction (37–39). Additionally, plant-derived compounds, such as *Ginkgo biloba* extract, have been shown to enhance ocular blood supply and protect the optic nerve. The mechanisms of action include vasodilation, reduced blood viscosity, decreased platelet aggregation, and antioxidant effects, all of which contribute positively to the recovery of visual function in glaucoma patients (40, 41). Our findings support the use of these agents in glaucoma management and provide a theoretical basis for their mechanisms. However, further research is needed to verify their detailed mechanisms and efficacy.

### Limitations

This study has several limitations. First, the sample size was small, which may not adequately represent the broader glaucoma population. Future research with a larger sample size is necessary for further validation. Second, the 6-month follow-up period may be insufficient; longer-term observations are needed to better understand the impact of IOP reduction on retinal microcirculation and visual field changes. Third, due to the limited sample size, we did not stratify patients into surgically controlled and drug-controlled groups, making it impossible to fully assess the impact of medications on blood flow changes. Some anti-glaucoma medication, such as β-adrenoreceptor blockers and prostaglandin derivatives, may indeed improve retinal vascular density (VD) (42, 43) in glaucoma patients. In this study, prostaglandin derivatives and β-adrenoreceptor blockers were frequently used in surgical patients before trabeculectomy and medicine-treated patients before enrollment, which could potentially improve baseline retinal VD. Moreover, the medicine-treated patients after enrollment only added carbonic anhydrase inhibitor and adrenergic α2 receptor agonists, which barely affect retinal blood vessels (22–24). However, after discontinuing all medications following trabeculectomy in surgical patients and continuing IOP-lowering treatment in medicine-treated patients, an overall VD increase in the optic disc region was still observed at the 6-month follow-up, suggesting a closer relationship between changes in VD and IOP reduction.

## Conclusion

Effective IOP lowering can improve microcirculation in the peripapillary and macular regions of patients with PACG or CPACG, particularly in the peripapillary region. This improvement may lead to a certain degree of enhanced visual function, although it does not appear to alter the structure of the optic nerve. The relationship between structural changes in the optic nerve and visual function may not be strictly linear. The primary factor contributing to visual function improvement may be the enhancement of peripapillary and macular microcirculation in response to IOP decline, rather than IOP reduction alone. These findings suggest that treatments aimed at improving microcirculation could be beneficial for visual function recovery in glaucoma patients, in conjunction with effective IOP-lowering therapies.

## Data Availability

The original contributions presented in the study are included in the article/Supplementary material, further inquiries can be directed to the corresponding author.

## Supplementary material

Supplementary Figure 1 Changes in optic nerve structure and function following treatment. **(A)** The C/D ratio exhibited minimal changes throughout the follow-up period. **(B)** RNFL thickness showed a gradual decrease over time following treatment. **(C)** FAZ size fluctuated throughout the treatment period. RNFL, retinal nerve fiber layer; FAZ, foveal avascular zone.
