# Supplementary figures and images for: The strong correlation between visual function improvement and retinal microcirculation enhancement in glaucoma
Source: Front Med (Lausanne). 2025 Mar 19;12:1537741. doi: 10.3389/fmed.2025.1537741 (PMC11961893; doi:10.3389/fmed.2025.1537741)

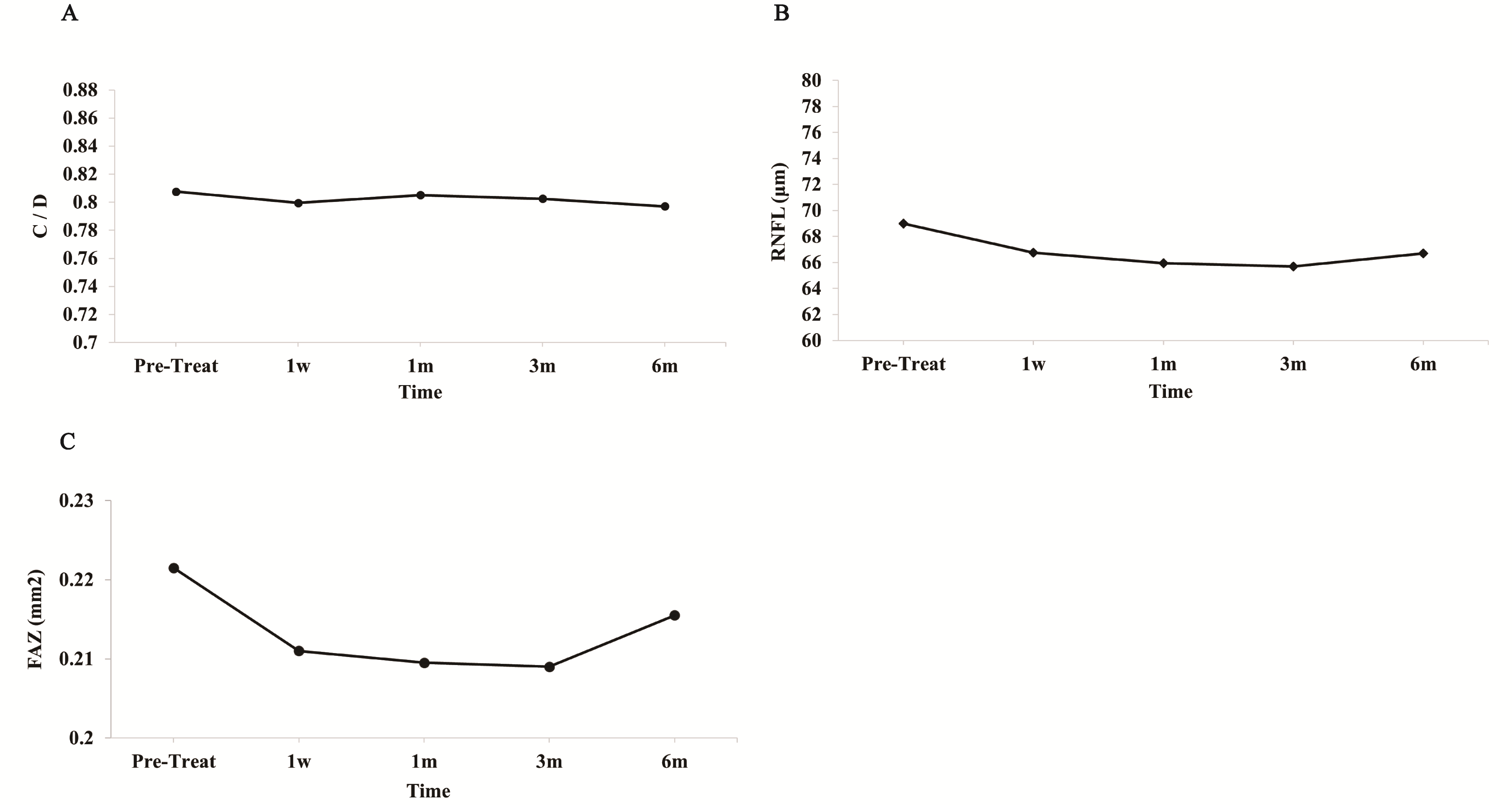

Supplement: Supplementary Figure 1 — Changes in optic nerve structure and function following treatment. (A) The C/D ratio exhibited minimal changes throughout the follow-up period. (B) RNFL thickness showed a gradual decrease over time following treatment. (C) FAZ size fluctuated throughout the treatment period. RNFL, retinal nerve fiber layer; FAZ, foveal avascular zone. [file Image_1.TIF]
