# Supplementary material for: The strong correlation between visual function improvement and retinal microcirculation enhancement in glaucoma
Source: Front Med (Lausanne). 2025 Mar 19;12:1537741. doi: 10.3389/fmed.2025.1537741 (PMC11961893; doi:10.3389/fmed.2025.1537741)
Supplement: Supplementary file 2 [file Table_1.DOCX]

| Patient | Type | PGs | Period | β-blocks | Period | α2-agonists | Period | CAIS | Period | CD | Period |
| --- | --- | --- | --- | --- | --- | --- | --- | --- | --- | --- | --- |
| 1 | POAG | + | 5m | + | 1m | + | 6m | + | 4m | - | N/A |
| 2 | CPACG | - | N/A | + | 5m | + | 5m | + | 3m | + | 3w |
| 3 | CPACG | + | 1m | - | N/A | + | 2m | + | 2m | - | N/A |
| 4 | CPACG | + | 2w | + | 1m | + | 3w | + | 1m | + | 3w |
| 5 | CPACG | + | 3w | + | 2m | + | 2m | + | 1m | - | N/A |
| 6 | CPACG | - | N/A | + | 7m | + | 1m | + | 5m | + | 2w |
| 7 | CPACG | + | 2w | - | 1m | + | 3w | + | 1m | - | N/A |
| 8 | CPACG | + | 5m | + | 6m | + | 9m | + | 1y | + | 3w |
| 9 | POAG | + | 8m | + | 6m | + | 7m | + | 6m | - | N/A |

**Table 1. The types and duration of medication used for surgical patients before surgery.**

POAG: primary open-angle glaucoma; CPACG: Chronic primary angle-closure glaucoma; PGs: prostaglandin derivatives; β-blocks: β-adrenoreceptor blockers; α2-agonists: adrenergic α2 receptor agonists; CAIS: carbonic anhydrase inhibitor; CD: Cholinomimetic drug; m: month; N/A: Not Applicable; w: week; y: year.

+: Drugs were used before enrollment. -: Drugs were not used before enrollment.

**Table 2. The types and duration of medication used for medicine-treated patients before enrollment.**

| Patient | Type | PGs | Period | β-blocks | Period | α2-agonists | Period | CAIS | Period |
| --- | --- | --- | --- | --- | --- | --- | --- | --- | --- |
| 1 | CPACG | - | N/A | + | 2w | - | N/A | - | N/A |
| 2 | POAG | + | 1m | - | N/A | - | N/A | - | N/A |
| 3 | POAG | + | 2m | - | N/A | - | N/A | - | N/A |
| 4 | CPACG | - | N/A | + | 3w | - | N/A | - | N/A |
| 5 | POAG | + | 1m | - | N/A | - | N/A | - | N/A |
| 6 | CPACG | - | N/A | - | N/A | - | N/A | + | 1m |
| 7 | POAG | + | 3w | - | N/A | - | N/A | - | N/A |
| 8 | POAG | + | 1m | - | N/A | - | N/A | - | N/A |
| 9 | POAG | - | N/A | - | N/A | + | 3w | - | N/A |
| 10 | CPACG | - | N/A | + | 1m | - | N/A | - | N/A |
| 11 | POAG | + | 3m | - | N/A | - | N/A | - | N/A |

POAG: primary open-angle glaucoma; CPACG: Chronic primary angle-closure glaucoma; PGs: prostaglandin derivatives; β-blocks: β-adrenoreceptor blockers; α2-agonists: adrenergic α2 receptor agonists; CAIS: Carbonic anhydrase inhibitor; m: month; N/A: Not Applicable; w: week.

+: Drugs were used before enrollment. -: Drugs were not used before enrollment.
